# SU11652 Inhibits tyrosine kinase activity of FLT3 and growth of MV-4-11 cells

**DOI:** 10.1186/1756-8722-5-72

**Published:** 2012-12-06

**Authors:** Yao Guo, Yun Chen, Xuesong Xu, Xueqi Fu, Zhizhuang Joe Zhao

**Affiliations:** 1 Edmond H. Fischer Signal Transduction Laboratory, College of Life Sciences, Jilin University, Changchun, China; 2 Department of Pathology, University of Oklahoma Health Sciences Center, Oklahoma City, Oklahoma, 73104, USA; 3 Clinical Laboratory, China Japan Union Hospital, Jilin University, Changchun, China

**Keywords:** Tyrosine kinase, FLT3, Inhibitor screening, SU11652, Acute myeloid leukemia

## Abstract

**Background:**

FLT3-ITD and FLT3-TKD mutations are frequently found in acute myeloid leukemia (AML). This makes tyrosine kinase FLT3 a highly attractive target for therapeutic drug development. However, effective drugs have not yet emerged. This study is intended to identify and to characterize new FLT3 inhibitors.

**Methods:**

By using the protein substrate GST-FLT3S to analyze kinase activity of recombinant proteins carrying the catalytic domain of wild type and mutant forms of FLT3, we screened a chemical library containing 80 known protein kinase inhibitors. We identified SU11652 as a potent FLT3 inhibitor and further employed FLT3-ITD-positive MV- 4–11 cells to study its effects on cell growth, apoptosis, cell cycles, and cell signaling.

**Results:**

SU11652 strongly inhibited the activity of wild type, D835Y, and D835H mutant forms of FLT3 with IC_50_ values of 1.5, 16, and 32 nM, respectively. It effectively blocked the growth of FLT3-ITD -positive MV-4-11 cells at nanomolar concentrations but exhibited much less effects on several other cells which do not carry mutations of FLT3. SU11652 inhibited growth of MV-4-11 cells by inducing apoptosis, causing cell cycle arrest, and blocking activation of the ERK, Akt, and STAT signaling pathways.

**Conclusion:**

SU11652 is a potent FLT3 inhibitor which selectively targets FLT3-ITD-positive cells. It should serve as a good candidate for development of therapeutic drugs to treat AML.

## Background

Acute myeloid leukemia (AML) is the most common malignant myeloid disorder in adults and is featured by abnormal differentiation and proliferation of hematopoietic progenitor cells of the myeloid lineage [1]. The median age of AML patients at diagnosis is 67 years, and it is estimated that 13,780 (7,350 men and 6,430 women) will be diagnosed with and 10,200 will die of the disease in 2012 in USA [2]. Currently, there is no effective cure for the disease. With standard chemotherapy, the 3-year event-free survival rate of patients with favorable cytogenetics is 60%; however, the median survival of patients with unfavorable cytogenetics is only 6 months [1,3]. Therefore, novel therapies with higher efficacy and fewer side effects are badly needed. With the increased understanding of the pathogenesis of AML, several molecular lesions have been identified, which allows development of targeted drugs [4]. Among them, FMS-like tyrosine kinase 3 (FLT3) is one of the most attractive targets, and related drug therapies are currently under development [5,6].

FLT3 is a receptor tyrosine kinase mainly expressed in early myeloid and lymphoid progenitors, and it plays a role in the development of hematopoietic progenitor cells [7]. Mutations of FLT3 have been found in about 30% of AML patients [8]. Among these mutations, the most common type is FLT3-ITD caused by internal tandem duplication (ITD) in the juxtamembrane domain, and the less common FLT3-TKD type involves point mutations in the tyrosine kinase domain (TKD). These mutations lead to constitutive activation of FLT3 and result in uncontrolled proliferation of leukemia cells [9], and they are strongly associated with poor prognosis in AML patients [10]. Since mutations found in AML cause activation of FLT3, FLT3 has become an obvious target for anticancer drugs. Up to date, several small molecule inhibitors and monoclonal antibodies against FLT3 have been developed [11-14]. Clinical trials have shown the efficacy of some FLT3 inhibitors in treatment of AML, but the responses are incomplete and often limited by the acquired resistance during the treatment [15-17]. For off-label use, sorafenib, which was initially identified as an inhibitor of VEGFR/PDGFR tyrosine kinases and Raf kinases, is the only FDA-approved anticancer drug targeting FLT3 [18]. Needless to say, identification of novel FLT3 inhibitors with higher potency is required.

In this study, by screening a protein kinase inhibitor library using a protein substrate we recently developed [19], we identified SU11652 as a potent FLT3 inhibitor. We further performed cell-based assays to demonstrate its inhibitory selectivity and potency for FLT3-ITD-positive cells and the cellular and molecular mechanism underlying its action. We believe that SU11652 should serve as a good candidate for development of targeted drugs to treat AML.

## Results and discussion

### SU11652 is a potent FLT3 inhibitor

**Figure 1:**
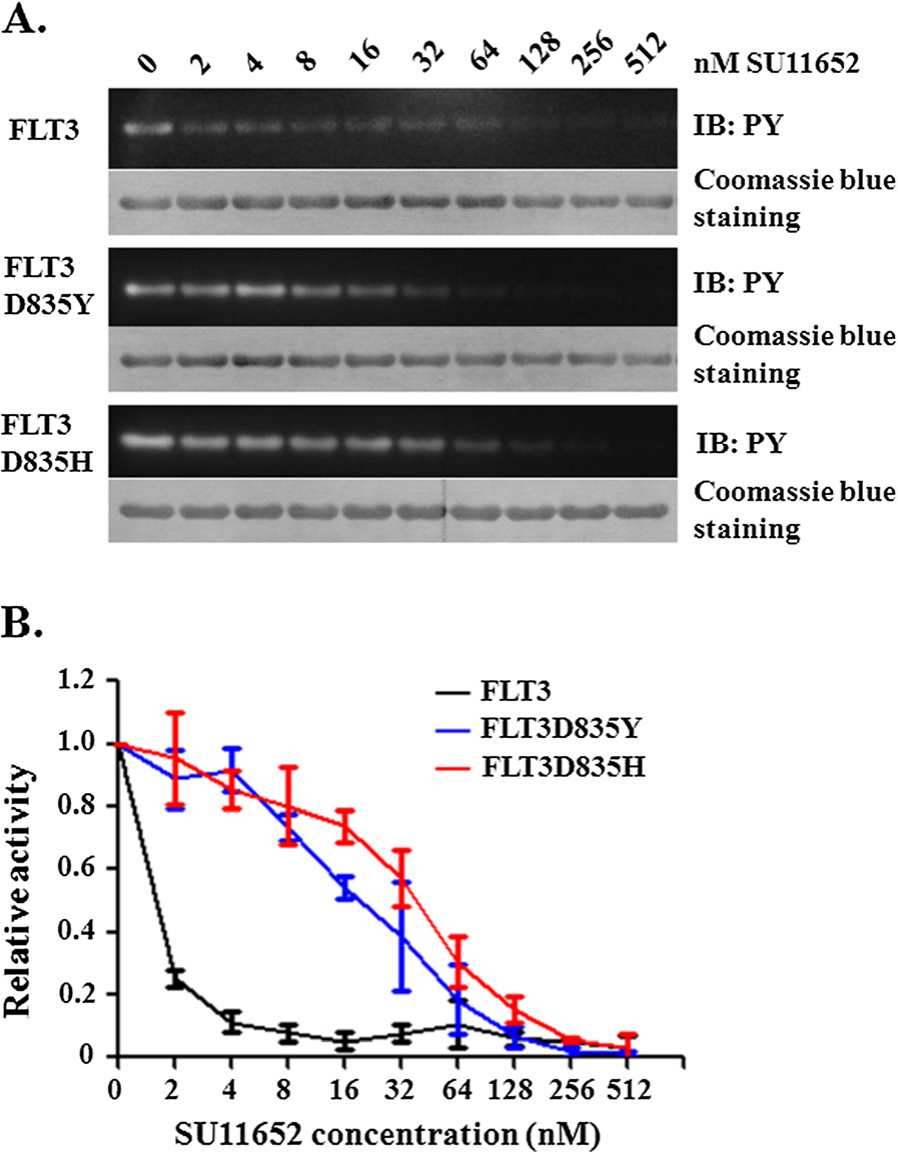
**SU11652 is a potent FLT3 inhibitor. A.** Tyrosine kinase activity of recombinant FLT3, FLT3D835H, and FLT3 D835Y was analyzed with GST-FLT3S as the substrate in the presence of various concentrations of SU11652. Tyrosine phosphorylation of GST-FLT3S was detected by using anti-phosphotyrosine antibody PY20, and its protein level, by Coomassie blue staining. **B.** The relative kinase activity was calculated based on the density of the western blot bands normalized to the control group. Error bars denote standard deviation (n = 3).

In our previous studies, we developed a system to analyze kinase activity of FLT3 by using a protein substrate [19]. We now employed the assays to screen chemical libraries for identification of FLT3 inhibitors. The library that we chose for this study is the InhibitorSelect™ 96-Well Protein Kinase Inhibitor Library I from Calbiochem. The library consists of 80 cell-permeable protein kinase inhibitors. The compounds were supplied at a concentration of 10 mM in dimethyl sulfoxide (DMSO). Our initial screening was performed with 3 μM and then 0.3 μM of each inhibitor. This led to identification of several compounds that exhibited strong inhibitory effects on FLT3 and its FLT3D835Y and FLT3 D835H mutant forms (data not shown). One of the compounds, SU11652, was further characterized. Figure 1 shows the dose responses. SU11652 inhibited the kinase activity of wild type FLT3 with an IC_50_ value of ~1.5 nM. It displayed relatively lower potency toward the FLT3D835Y and FLT3D835H mutants with IC_50_ values of 16 and 32 nM, respectively. SU11652 is a sunitinib-like oxindole inhibitor and has been found as an inhibitor of PDGFRβ, VEGFR2, FGFR1, Flk-1, and cKit with IC_50_ or Ki values of 3–500 nM [20,21]. Our study now suggests that it inhibits FLT3 with even higher potency. It is interesting to note that the D835Y and D835H mutant forms of FLT3 are less sensitive to SU11652 than the wild type FLT3. This is reminiscent of data obtained with two other known FLT3 inhibitors, namely, sorafenib and AC220 (quizartinib) [15]. On the other hand, FLT3-ITD mutants contain the wild type kinase domain and should be highly sensitive to inhibition by SU11652. Therefore, in the clinical applications, SU11652 would be more suitable for patients with FLT3-ITD mutations than those with FLT3-TDK mutations.

### SU11652 inhibits the growth of FLT3-ITD-positive cells

**Figure 2:**
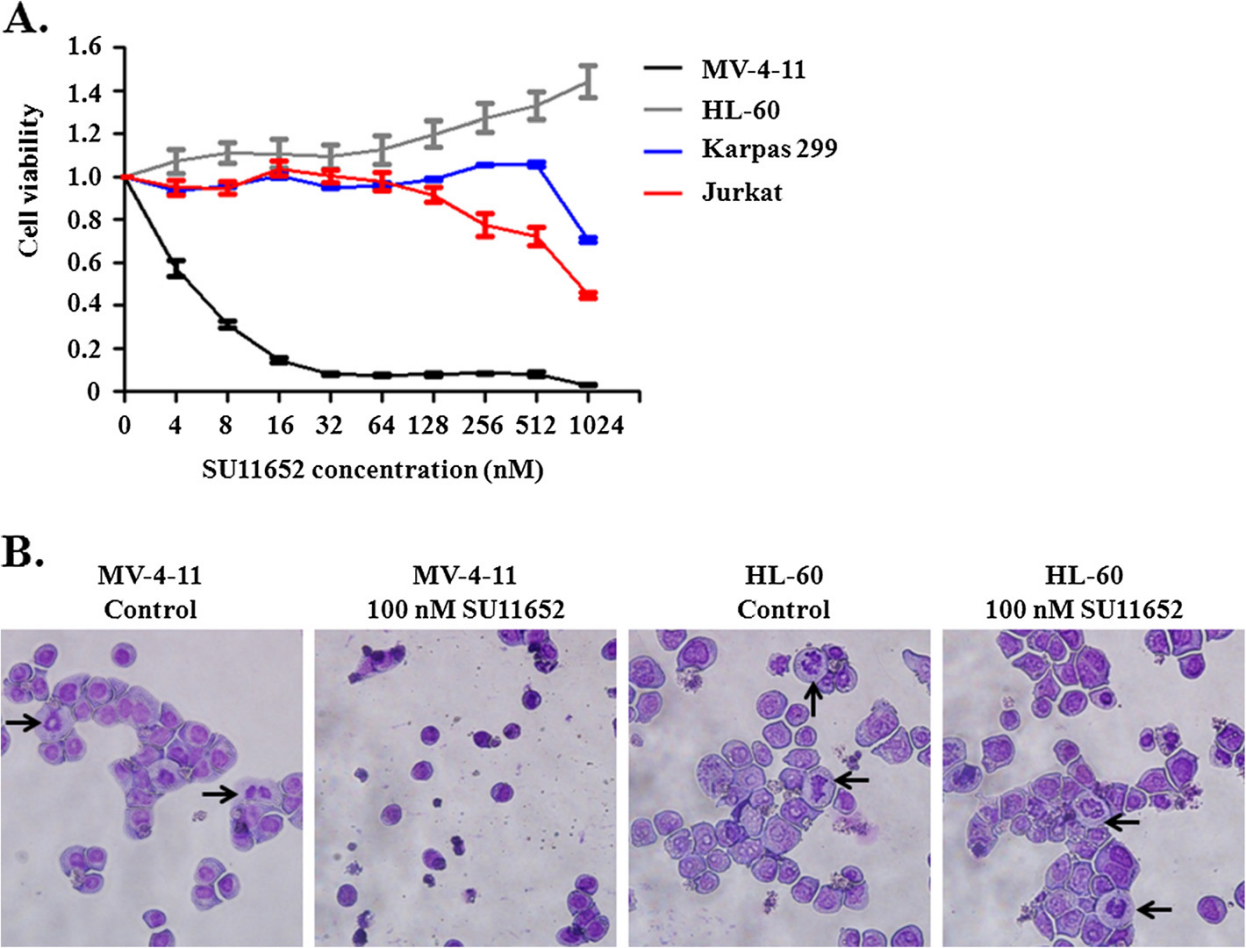
**SU11652 effectively inhibits the growth of MV**-**4**-**11 cells. A.** MV-4-11, HL-60, Karpas 299, and Jurkat cells were cultured in the presence of various concentrations of SU11652 for 48 hours. Cell viability was assessed by MTT assays. Error bars denote standard deviation (n = 3). **B.** Wright-Giemsa staining of MV-4-11 and HL-60 cells treated with 0 or 100 nM SU11652 for 48 h. Black arrows point to mitotic cells.

We further employed cell-based assays to verify the inhibitory effects of SU11652 on FLT3. For this purpose, the MV-4-11 cell line was employed. The cells were derived from biphenotypic B myelomonocytic leukemia and carry a FLT3-ITD mutation [22]. As expected, MTT assays revealed that MV-4-11 cells were highly sensitive to SU11652 with an IC_50_ value of ~5 nM (Figure 2A). In contrast, HL-60 acute promyelocytic leukemia, Jurkat acute T cell leukemia cells, and Karpas 299 anaplastic large cell lymphoma cells were hardly affected by the inhibitor at 500 nM. These cells do not carry FLT3 mutations. The data demonstrate that SU11652 specifically targets cells containing FLT3-ITD. It should be noted that Karpas 299 cells contain a mutation of tyrosine kinase Alk and a p53 mutation [23,24]. Apparently, SU11652 is selective for tyrosine kinases mutated in cancer cells. The inhibitory effects of SU11652 on the growth of MV-4-11 cells were also demonstrated by Wright-Giemsa staining of cells fixed to glass slides by cytospin. As shown in Figure 2B, in comparison with the non-treated MV-4-11 cells, cells treated with 100 nM SU11652 were sparser and smaller, showing no mitotic cells but rather condensed nuclei and cell debris. As a control, HL-60 cells behaved normally showing regular morphology and many mitotic cells in the presence of 100 nM SU11652.

### SU11652 induces apoptosis and cell cycle arrest in MV-4-11 cells

**Figure 3:**
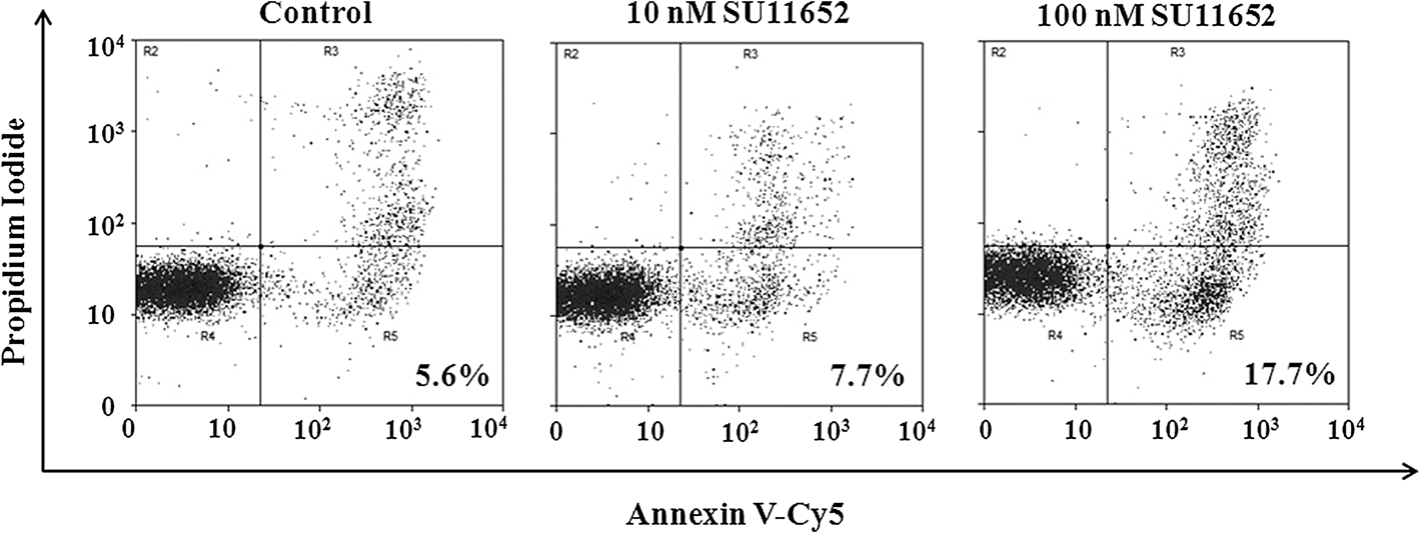
**SU11652 induces apoptosis of MV**-**4**-**11 cells.** MV-4-11 cells were incubated with 0, 10 and 100 nM SU11652 for 24 hours. Cells were stained with Cy5-labeled annexin V and propidium iodide, followed by analyses with a flow cytometry. Percentages of annexin V-positive and propidium iodide-negative cells, that is, apoptotic cells, are indicated.

**Figure 4:**
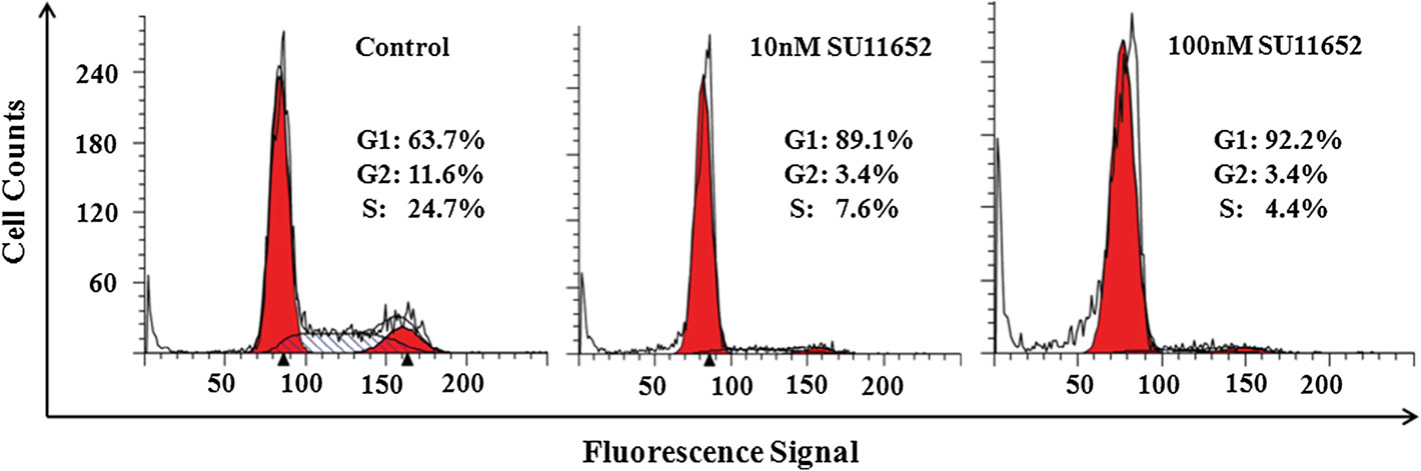
**SU11652 induces cell cycle arrest of MV**-**4**-**11 cells.** MV-4-11 cells were incubated with 0, 10, and 100 nM SU11652 for 24 hours. Cells were fixed with ethanol and stained with propidium iodide before flow cytometric analysis. Percentages of cells in G1, S, and G2 phases were calculated by using the ModFit software.

To reveal further how SU11652 inhibits the growth of MV-4–11 cells, we conducted apoptosis assays and cell cycle analyses. Apoptosis was demonstrated by staining with Annexin V and propidium iodide. As shown in Figure 3, the percentage of Annexin V-positive and propidium iodide-negative cells was increased following SU11652 treatment, reaching 17.7% at 100 nM, indicating induction of apoptotic cell death. The effects of SU11652 on the cell cycle were even more prominent. As shown in Figure 4, treatment of MV-4-11 cells with 10 nM SU11652 reduced S phase cells from 24.7% to 7.6%, accompanied by comparable reduction in the percentages of G2 phase cells. Together, the data indicate that SU11652 induces both apoptosis and cell cycle arrest of MV-4-11 cells.

### SU11652 inhibits FLT3 downstream signaling in MV-4-11 cells

**Figure 5:**
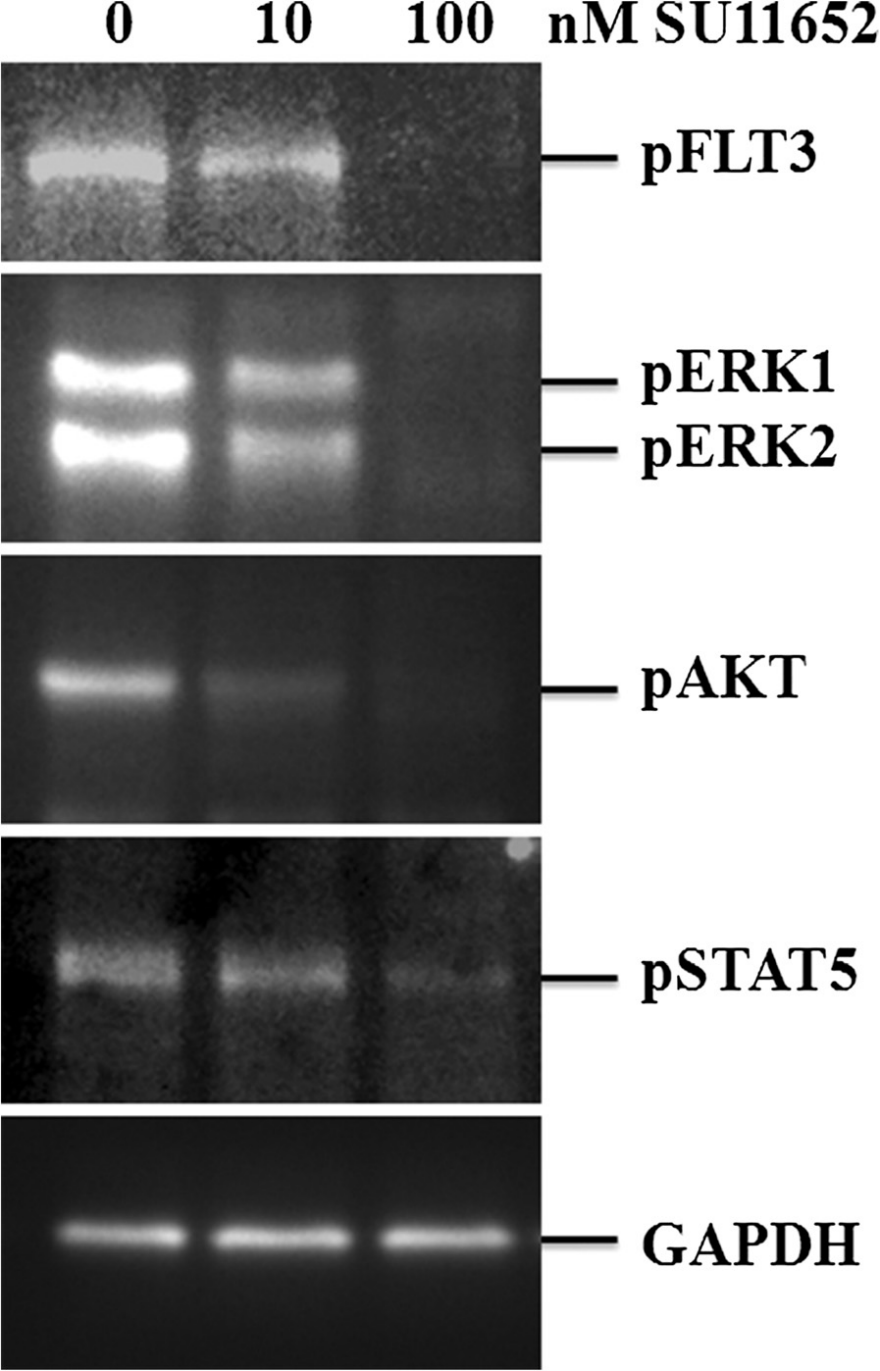
**SU11652 blocks activation of signal transduction pathways downstream of FLT3.** MV-4-11 cells were treated with the indicated concentrations of SU11652 for 24 hours. Cell extracts were subjected to western blot analyses with antibodies against phosphorylated forms of FLT3 (pY591), ERK1/2 (pT202/pY204), Akt (pS473), and STAT5 (pY694). Equal protein loading was demonstrated by blotting with house-keeping gene product GAPDH.

As a growth factor receptor, FLT3 turns on various signaling pathways to regulate cell growth and proliferation [9]. These include the Ras/MAPK, PI3K/Akt, and JAK/STAT pathways. Mutations of FLT3 are known to cause constitutive activation of FLT3 kinase activity and downstream signaling components [9]. To see if the inhibition of FLT3 kinase activity by SU11652 also blocks its downstream signal transduction events, we used phospho-specific antibodies to detect activation of ERK1/2, Akt, and STAT5 in MV-4-11 cells. Figure 5 shows a dose-dependent reduction in the phosphorylation levels of FLT3, ERK1/2, Akt, and STAT5 in MV-4-11 cells treated with SU11652. This indicates that the activation of these downstream signaling proteins in MV-4–11 is blocked by SU11652, thereby providing a molecular mechanism for the inhibition of cell growth by the inhibitor.

## Conclusions

By screening a chemical library with an effective FLT3 kinase assay system, we have identified SU11652 as a potent inhibitor of FLT3 with an IC_50_ value of 1.5 nM. More importantly, our *in vitro* cell-based assays demonstrated that SU11652 selectively inhibited the growth of FLT3-ITD-positive MV-4-11cells with equivalent potency. Furthermore, we showed that SU11652 induced apoptosis, caused cell cycle arrest, and blocked FLT3 downstream signaling transduction. FLT3 is an obvious target for therapeutic drugs to AML, but no effective drug has emerged. Our study provides a new candidate. Considering the potency and selectivity of SU11652 according to biochemical and cell-based assays, further preclinical study with animal models and clinical studies with FLT3-ITD -positive AML patients appears to be well warranted.

## Methods

### Materials

InhibitorSelect Protein Kinase Library I containing 80 protein kinase inhibitors including SU11652 was purchased from Calbiochem (CA, USA). Monoclonal anti-phosphotyrosine antibody PY20 was from BD Biosciences (CA, USA), while antibodies against pFLT3 (pY591), pERK1/2(pT202/pY204), pAKT(pS473), and pSTAT5(pY694) were from Cell Signaling Technology (MA, USA). MV-4–11, HL-60, and Jurkat cell lines were obtained from ATCC (VA, USA). Karpas 299 cells were kindly provided by Yi Zhao (University of Southern California, [23]). MV-4-11 cells were cultured in Iscove’s Modified Dulbecco’s Medium containing 10% fetal bovine serum, and the rest of cells were maintained in RPMI medium supplemented with 10% fetal bovine serum.

### FLT3 kinase activity assays and inhibitor screening

Protein kinase activity assays and inhibitor screening were performed as previously described [19,25]. The FLT3 substrate GST fusion protein GST-FLT3S was purified from *E. coli* cells by using a glutathione-Sepharose column, and recombinant proteins containing the catalytic domain of wild type FLT3 and its D835H and D835Y mutant forms were isolated from recombinant baculovirus-infected Sf9 insect cells by using the NTA-Ni resin [19]. Phosphorylation of GST-FLT3S by isolated FLT3 tyrosine kinases was carried out in a reaction buffer containing 25 mM Tris–HCl (pH 7.5), 10 mM MgCl_2_, 0.2 mM adenosine 5^′^-triphosphate, and 2 mM dithiothreitol in the presence of various concentrations of protein kinase inhibitors. The level of GST-FLT3S tyrosine phosphorylation was determined by immunoblotting with anti-phosphotyrosine antibody PY20 followed by horseradish peroxidase-conjugated secondary antibody. Detection and quantification of enhanced chemiluminescence signals were done by using FluorChem SP imaging system from Alpha Innotech [26].

### Cell viability assays

MV-4-11, HL-60, Karpas 299, and Jurkat cells were incubated with various concentrations of SU11652 for 48 hours. To measure the viability of cells, 0.5 mg/ml MTT (3-(4,5-dimethylthiazol-2-yl)-2,5-diphenyltetrazolium bromide) was added into the medium. After incubation at 37°C for 3 hours, the medium was removed by centrifugation and the precipitated dye was dissolved in 1 ml isopropanol containing 0.04 M HCl. Absorbance at 570 nm was then measured with a spectrophotometer.

### Apoptosis and cell cycle analyses

For apoptosis analysis, the cells were stained with Annexin V-Cy5 and propidium iodide (Biovision, CA, USA). To assess cell cycle arrest, the cells were fixed with ethanol overnight and then stained with propidium iodide in the presence of RNAse. Flow cytometric assays were performed by using a FACSCalibur flow cytometer (BD Biosciences) at the Flow and Image Cytometry Laboratory of University of Oklahoma Health Sciences Center.

### Cell signaling assays

Cells treated with SU11652 or the control solvent were extracted with a whole-cell extraction buffer containing 25 mM β-glycerophosphate (pH 7.3), 5 mM EDTA, 2 mM EGTA, 5 mM β-mercaptoethanol, 1% Triton X-100, 0.1 M NaCl, 1 mM sodium vanadate, and a protease inhibitor cocktail (Roche Applied Science, Indianapolis, IN, USA). Cell lysates were cleared by centrifugation in a microfuge at 13,000 g, and clear cell extracts containing equal amounts of total proteins were separated on SDS gels for western blot analyses with antibodies against pFLT3, pERK, pAKT, and pSTAT5.

## Abbreviations

AML: Acute myeloid leukemia; GST: Glutathione S-transferase.

## Competing interests

The authors declare no conflict of interests.

## Authors’ contributions

GY and YC performed the research experiments; XX designed the research; XF and ZJZ designed and supervised the research. All authors wrote and approved the manuscript.

## References

[B1] Estey E Döhner H Acute myeloid leukaemia Lancet 2006 368 1894 1907 10.1016/S0140-6736(06)69780-8 17126723

[B2] Howlader N Noone AM Krapcho M Neyman N Aminou R Altekruse SF Kosary CL Ruhl J Tatalovich Z Cho H Mariotto A Eisner MP Lewis DR Chen HS Feuer EJ Cronin KA SEER Cancer Statistics Review, 1975–2009 (Vintage 2009 Populations) Bethesda, MD: National Cancer Institute http://seer.cancer.gov/csr/1975_2009_pops09/.

[B3] Estey EH Acute myeloid leukemia: 2012 update on diagnosis, risk stratification, and management Am J Hematol 2012 87 89 99 10.1002/ajh.22246 22180162

[B4] Takahashi S Current findings for recurring mutations in acute myeloid leukemia J Hematol Oncol 2011 4 36 10.1186/1756-8722-4-36 21917154 PMC3180439

[B5] Swords R Freeman C Giles F Targeting the FMS-like tyrosine kinase 3 in acute myeloid leukemia Leukemia 2012 26 2176 2185 10.1038/leu.2012.114 22614177

[B6] Leung AY Man CH Kwong YL FLT3 inhibition-a moving and evolving target in acute myeloid leukaemia Leukemia 2012 10.1038/leu.2012.195 22797419

[B7] Stirewalt DL Radich JP The role of FLT3 in haematopoietic malignancies Nat Rev Cancer 2003 3 650 665 10.1038/nrc1169 12951584

[B8] Gregory TK Wald D Chen Y Vermaat JM Xiong Y Tse W Molecular prognostic markers for adult acute myeloid leukemia with normal cytogenetics J Hematol Oncol 2009 2 23 10.1186/1756-8722-2-23 19490647 PMC2700131

[B9] Takahashi S Downstream molecular pathways of FLT3 in the pathogenesis of acute myeloid leukemia: biology and therapeutic implications J Hematol Oncol 2011 4 13 10.1186/1756-8722-4-13 21453545 PMC3076284

[B10] Schlenk RF Döhner K Krauter J Fröhling S Corbacioglu A Bullinger L Habdank M Späth D Morgan M Benner A Schlegelberger B Heil G Ganser A Döhner H German-Austrian Acute Myeloid Leukemia Study Group Mutations and treatment outcome in cytogenetically normal acute myeloid leukemia N Engl J Med 2008 358 1909 1918 10.1056/NEJMoa074306 18450602

[B11] Hofmann M Große-Hovest L Nübling T Pyż E Bamberg ML Aulwurm S Bühring HJ Schwartz K Haen SP Schilbach K Rammensee HG Salih HR Jung G Generation, selection and preclinical characterization of an Fc-optimized FLT3 antibody for the treatment of myeloid leukemia Leukemia 2012 26 1228 1237 10.1038/leu.2011.372 22289926

[B12] Zhu X Ma Y Liu D Novel agents and regimens for acute myeloid leukemia: 2009 ASH annual meeting highlights J Hematol Oncol 2010 3 17 10.1186/1756-8722-3-17 20416083 PMC2880983

[B13] Zarrinkar PP Gunawardane RN Cramer MD Gardner MF Brigham D Belli B Karaman MW Pratz KW Pallares G Chao Q Sprankle KG Patel HK Levis M Armstrong RC James J Bhagwat SS AC220 is a uniquely potent and selective inhibitor of FLT3 for the treatment of acute myeloid leukemia (AML) Blood 2009 114 2984 2992 10.1182/blood-2009-05-222034 19654408 PMC2756206

[B14] O’Farrell AM Abrams TJ Yuen HA Ngai TJ Louie SG Yee KW Wong LM Hong W Lee LB Town A Smolich BD Manning WC Murray LJ Heinrich MC Cherrington JM SU11248 is a novel FLT3 tyrosine kinase inhibitor with potent activity in vitro and in vivo Blood 2003 101 3597 3605 10.1182/blood-2002-07-2307 12531805

[B15] Smith CC Wang Q Chin CS Salerno S Damon LE Levis MJ Perl AE Travers KJ Wang S Hunt JP Zarrinkar PP Schadt EE Kasarskis A Kuriyan J Shah NP Validation of ITD mutations in FLT3 as a therapeutic target in human acute myeloid leukaemia Nature 2012 485 260 263 10.1038/nature11016 22504184 PMC3390926

[B16] Moore AS Faisal A Gonzalez de Castro D Bavetsias V Sun C Atrash B Valenti M de Haven Brandon A Avery S Mair D Mirabella F Swansbury J Pearson AD Workman P Blagg J Raynaud FI Eccles SA Linardopoulos S Selective FLT3 inhibition of FLT3-ITD(+) acute myeloid leukaemia resulting in secondary D835Y mutation: a model for emerging clinical resistance patterns Leukemia 2012 26 1462 1470 10.1038/leu.2012.52 22354205 PMC3523391

[B17] Kindler T Lipka DB Fischer T FLT3 as a therapeutic target in AML: still challenging after all these years Blood 2010 116 5089 5102 10.1182/blood-2010-04-261867 20705759

[B18] Pratz KW Levis MJ Bench to Bedside Targeting of FLT3 in Acute Leukemia Curr Drug Targets 2010 11 781 789 10.2174/138945010791320782 20370649 PMC3023996

[B19] Chen Y Guo Y Han J Ho WT Li S Fu X Zhao Z Generation and characterization of a highly effective protein substrate for analysis of FLT3 activity J Hematol Oncol 2012 5 39 10.1186/1756-8722-5-39 22800464 PMC3419602

[B20] Liao AT Chien MB Shenoy N Mendel DB McMahon G Cherrington JM London CA Inhibition of constitutively active forms of mutant kit by multitargeted indolinone tyrosine kinase inhibitors Blood 2002 100 585 593 10.1182/blood-2001-12-0350 12091352

[B21] Sun L Liang C Shirazian S Zhou Y Miller T Cui J Fukuda JY Chu JY Nematalla A Wang X Chen H Sistla A Luu TC Tang F Wei J Tang C Discovery of 5-[5-fluoro-2-oxo-1,2- dihydroindol-(3Z)-ylidenemethyl]-2,4- dimethyl-1H-pyrrole-3-carboxylic acid (2-diethylaminoethyl)amide, a novel tyrosine kinase inhibitor targeting vascular endothelial and platelet-derived growth factor receptor tyrosine kinase J Med Chem 2003 46 1116 1119 10.1021/jm0204183 12646019

[B22] Quentmeier H Reinhardt J Zaborski M Drexler HG FLT3 mutations in acute myeloid leukemia cell lines Leukemia 2003 17 120 124 10.1038/sj.leu.2402740 12529668

[B23] Hsu FY Johnston PB Burke KA Zhao Y The Expression of CD30 in Anaplastic Large Cell Lymphoma Is Regulated by Nucleophosmin-Anaplastic Lymphoma Kinase–Mediated JunB Level in a Cell Type–Specific Manner Cancer Res 2006 66 9002 9008 10.1158/0008-5472.CAN-05-4101 16982741

[B24] Zhao W Du Y Ho WT Fu X Zhao ZJ JAK2V617F and p53 mutations coexist in erythroleukemia and megakaryoblastic leukemic cell lines Experimental Hematology & Oncology 2012 1 15 10.1186/2162-3619-1-15 23210734 PMC3514099

[B25] Li Z Xu M Xing S Ho WT Ishii T Li Q Fu X Zhao ZJ Erlotinib effectively inhibits JAK2V617F activity and polycythemia vera cell growth J Biol Chem 2007 282 3428 3432 17178722 10.1074/jbc.C600277200 PMC2096634

[B26] Li Z Xing S Wang S Ho WT Zhao ZJ Characterization of a highly effective protein substrate for analysis of JAK2(V617F) Activity Exp Hematol 2007 35 1624 1632 10.1016/j.exphem.2007.07.003 17764811 PMC2128699

